# Experimental Study of Drilling Damage Outcomes in Hybrid Composites with Waste Micro-Inclusions

**DOI:** 10.3390/ma16237325

**Published:** 2023-11-24

**Authors:** Luis M. P. Durão, João E. Matos, João Alves, Sérgio Moni Ribeiro Filho, Túlio H. Panzera, Fabrizio Scarpa

**Affiliations:** 1 Instituto de Ciência e Inovação em Engenharia Mecânica e Engenharia Industrial (INEGI), 4200-465 Porto, Portugal; 2 Instituto Superior de Engenharia do Porto (ISEP), Instituto Politécnico do Porto (IPP), Rua Dr. António Bernardino de Almeida, 4249-015 Porto, Portugal; jem@isep.ipp.pt (J.E.M.); 1140629@isep.ipp.pt (J.A.); 3 Centre for Innovation and Technology in Composite Materials—CITeC, Department of Mechanical Engineering, Federal University of São João del Rei—UFSJ, São João del Rei 36301-360, MG, Brazil; sergiolmrf@gmail.com (S.M.R.F.); panzera@ufsj.edu.br (T.H.P.); 4 Bristol Composites Institute (ACCIS), University of Bristol, Bristol BS8 1TR, UK; f.scarpa@bristol.ac.uk

**Keywords:** hybrid composites, waste reuse, drilling, flexural testing

## Abstract

Composite materials are used in a substantial number of products. Environmental concerns highlight the need for the inclusion of recovered waste in their formulation, thus reducing their carbon footprint. These solutions raise the need to confirm the mechanical characteristics of these materials, avoiding unwanted failures. In this work, the authors present an experimental study on the drilling effects on fibrous–particulate hybrid composites made of glass/carbon fabrics and three different micro-inclusions: silica particles, recycled carbon fibre powder and cement. The mechanical features of the plates are confirmed by thrust force monitoring during drilling and by flexural testing. The range of results confirm the mechanical outcomes due to machining. The plates with monolithic carbon fabric or with carbon fabric plies in the outer plies returned higher mechanical characteristics. The plates with micro-inclusions had enhanced the flexural strength by 23% and 10%, in 40% and 60% fabric plates, respectively. The results demonstrate that the use of alternative formulations with micro-inclusions from recovered waste can contribute both to the reduction of the mechanical degradation of drilled hybrid composites and to environmental purposes by avoiding the increase in landfill waste.

## 1. Introduction

Polymeric matrix composites, or fibre-reinforced polymers (FRP), have registered a considerable increase in their use, mainly since the second half of the past century. Their advantages are related to their low specific weight, good mechanical strength and stiffness. The continuous decrease in the price of materials and processing costs, together with the increase in reliability, have led to permanent innovation and to new applications. As the main advantages of composite materials lead to this extensive usage, a major drawback has arisen, which is related to the problem of recyclability at the end of their useful life. A possible alternative, as long as mechanical strength is not playing a major role, is the incorporation of particles in the formulation of composites.

FRPs are, by definition, a combination of two macro constituents—matrix and reinforcement—that are insoluble in each other [1]. Thus, in FRP parts the matrix is responsible for the bulk form of the part and chemical protection of the fibres, while the reinforcement is responsible for the mechanical resistance. Parts are designed in a way to take advantage of the fibre distribution to enhance mechanical resistance but, due to assembly or design purposes, they usually exhibit stress concentrations resulting from required assembly holes or imposed section changes. In the most common FRP parts only one matrix material and one reinforcement fibre are used. If there is more than one type of reinforcement, the composite is known as a hybrid. The need to address environmental issues, in addition to representing an option for recycling by-products from other industries, creates an opportunity for the incorporation of particles from discarded FRP parts as an alternative to the mere disposal of these parts in landfills [2]. Another foreseen advantage is the contribution to the overall demand for the reduction of carbon emissions. Different approaches are possible regarding these environmental concerns. In [3] Ribeiro Filho et al. presented a study on hybrid bio-composites reinforced with sisal–glass fibres and Portland cement particles, showing that the inclusion of cement particles had enhanced the flexural properties of the hybrid composites and that the stacking sequence strongly affected their mechanical properties. Gemi et al. [4] experimentally analysed the behaviour of pultruded GFRP composite beams infilled with hybrid fibre-reinforced concrete under four-point flexural loading. Furthermore, the authors analysed and then developed a numerical model to show the positive outcomes of this solution. In [2], Oliveira et al. presented a novel hybrid polymer composite made from sugarcane bagasse and discarded rubber particles, concluding that the mechanical and physical properties were substantially affected by the amount and size of the rubber particles and that the presence of sugarcane bagasse increased the compressive toughness of hybrid composites. The use of sustainable composites for transport applications had been presented in [5,6] regarding the development of alternative sustainable lightweight composites to reduce weight and costs in the automotive industry and in [7] for railways, considering the calculations of carbon emissions and energy consumption for the overall life cycle of sleepers, including recyclability.

Normally, parts are produced for supplementary grouping in structures; therefore, drilling is normally needed for enabling rapid assembly with rivets or bolts. One of the concerns when machining FRPs is related to the reinforcements, usually of an abrasive nature, causing rapid tool wear and deterioration of the machined surfaces. Typical damages after drilling are pushout delamination, peel-up delamination, fibre pull-out, burrs, splintering, swelling as well as some thermal damages due to matrix softening. When considering the drilling of composites, hole quality results are mainly fibre related [8]. The development of thrust forces during the drilling operation is accepted to be more related with the drill geometry, feed rate and spindle speed, as already demonstrated in several published studies by different authors, see [9,10,11,12,13,14,15,16,17,18,19,20], showing the importance of parameter selection for the minimization of machining damages. In [11] Khashaba suggested a new approach to the drilling of woven glass fibre-reinforced polymer (GFRP) composites: clamping the specimen with two support plates, thus controlling force at drill entry and exit sides. By using support plates, the thrust force was increased up to 8.3% compared to the unsupported conditions, but delaminations were decreased by about 65.5%. Gemi et al. [17,18] concluded that, regarding the stacking sequence, the one that considers a carbon layer between two glass layers (GCG) presented better performance in terms of mechanical properties and machinability characteristics. Particularly in [18], Gemi et al. were able to conclude that the stacking sequence considerably affects the damage modes and damage mechanisms of the composites. Moreover, lower delamination damage had occurred at lower feed rates and higher cutting speeds, as in the machining of composite plates. Moreover, a greater damage extension was formed in the GCG specimen. The drilling performance of stacked glass-carbon fibre-reinforced hybrid laminate composites was examined experimentally in [19]. In this work, Ergene et al. showed that the delamination can be decreased with higher cutting speeds or lower feed rates. A comprehensive study including the use of Taguchi techniques, analysis of variance (ANOVA) and the response surface method (RSM) seeking for cutting parameter optimization of hybrid fibre composite during drilling was conducted by Ozsoy et al. [20], concluding that a low cutting velocity and a low feed rate should be applied under dry conditions to reduce delamination. Alternative drilling strategies for delamination reduction have been studied. The use of cryogenic machining can reduce heat-generated damage and delamination [21] and the use of chilled air can also contribute to a decrease in delamination extension [22]. A different approach is suggested in [23], combining ultrasonic vibration and drilling to achieve higher hole quality.

The existence of a critical thrust force, *F_crit_*, for the onset of delamination, according to the model presented by Hocheng and Dharan [24], is well known, and shown in Equation (1):

(1) $$F_{crit}=\pi \frac{\sqrt{8G_{IC}E_{1}h^{3}}}{3\left(1-\nu_{12}^{2}\right)}$$

This critical thrust force is a function of the elastic modulus, *E*_1_, the Poisson ratio, *υ*_12_, the interlaminar fracture toughness in mode *I*, *G_Ic_* and the uncut plate thickness, *h*.

Concerns regarding the outcome of hybrid composites, namely with glass and carbon fibre as reinforcement, were studied by different authors either under tensile, compressive [25] or flexural loading [26,27], showing that hybridisation strategies can be helpful to balance costs, performance and reliability of composites for structural and lightweight applications.

The main goal of the present work is to demonstrate that waste particles can be used as reinforcement in hybrid composites, avoiding their disposal in landfills, and thus contributing to environmental purposes. The experimental array presented here is an addition to a previous study on ultrasonic pulse velocity and physical properties of the same set of hybrid composites [28]. By using the same planning regarding the stacking sequence, volume fraction and type of particle, this study concerns the outcomes of the drilling operation on the mechanical properties of hybrid composites by flexural testing. Reviews on the problem of delamination onset, assessment and suppression [29,30,31] were considered. In [29], Liu et al. summarised the most recent progresses in the mechanical drilling of composite laminates, covering drilling operations, drill bit geometry and materials, drilling-induced delamination and its suppressing approaches, thrust force and tool wear. Kumar and Singh [30] focused on the issues related to the conventional and unconventional machining of composite materials, more specifically on drilling. Most recently, a general review of drilling-induced delamination for composite laminates, including a delamination formation mechanism, delamination quantification methodologies and measurement technologies and delamination suppression strategies was presented by Geng et al. [31]. These reviews have helped to obtain a comprehensive view on mechanical drilling of composite laminates and on the planning of the experimental work. 

## 2. Materials and Methods

The experimental work aims to show the outcomes of drilling in hybrid composite plates made of glass/carbon fabrics and three diverse micro-inclusions like silica particles, recycled carbon microfibres and cement. See Table 1 and Table 2 for the factors considered, experimental levels and plate identification.

So, 2 plates for each formulation were produced, resulting in a total amount of 96 plates, with the dimensions of 100 × 100 × 1 mm^3^, prepared at the laboratories of Universidade Federal de São João del-Rei (UFSJR) in Brazil. 

**Figure 1:**
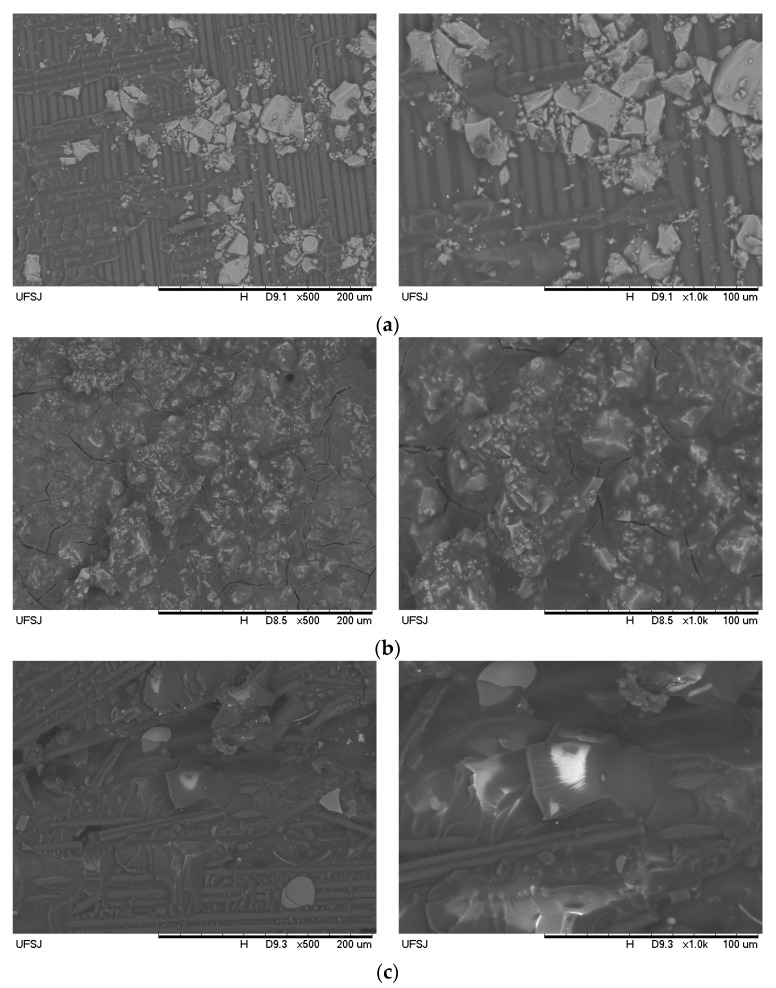
SEM images of some plates with fillers: (**a**) Silica (Plate 11); (**b**) Cement (Plate 17); (**c**) Carbon microfibres (Plate 23).

All the produced hybrid composites had 5 plies with different stacking sequences, 2 levels of reinforcement fraction and 4 types of particle inclusion (9% wt)—see Table 1 and Table 2. Plates were cured for 12 h under 0.8 kPa and 24 °C followed by a stage of 7 days at room temperature. The final thickness was 1 mm. In Figure 1, SEM images of some plates are presented, showing the diverse morphology that results from different particle inclusion. More details on the plates can be found in Ribeiro Filho et al. [28].

These combinations resulted in a total of 48 different conditions, grouped in 8 subsets of 6 conditions, to enable the simplest comparison of the results. These diverse subsets, divided according to the reinforcement volume fraction and particle type, are referred to as S1 to S8 (see also Table 2). The target of reusing production scrap or waste to contribute to a reduction of the amount of material that will end in landfills, thus addressing environmental issues, is a line of work that has been followed by the authors. As already discussed, this experimental work follows a previous characterization of mechanical and physical properties and their correlation to ultrasonic pulse velocity and physical properties. Further information regarding plate manufacturing, including physical properties as well as a description of the raw materials and a complete DOE and SEM analysis can be found in [28].

**Figure 2:**
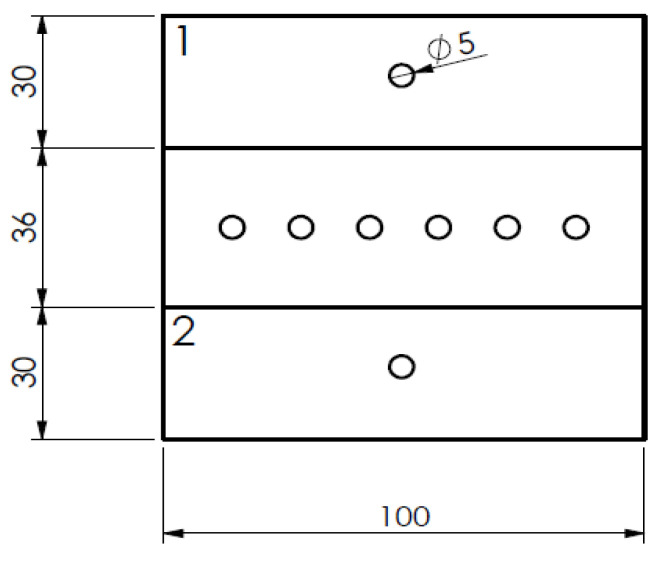
Drilling scheme of the plates. Plates numbered 1, 2 for tracking purpose.

Each of the 96 test plates was drilled according to the scheme shown in Figure 2, being the central section, with 6 holes, for the monitoring of thrust forces during drilling and the lateral holes to produce coupons for the flexural tests. The drilling sequence and subsequent plate cutting resulted in a total of 288 coupons (Figure 2).

**Figure 3:**
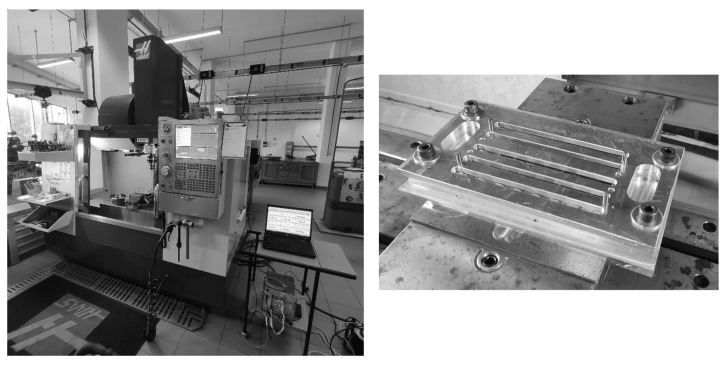
Experimental setup and support for plate positioning.

**Figure 4:**
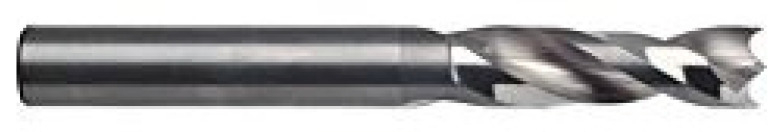
Brad point drill.

Drilling was performed on a HAAS VF-2 CNC and to enable a quick and steady positioning of the plates, a support was designed (see Figure 3 for experimental setup and plate support plate). The drilling parameters were set to 1120 rpm and a feed rate of 0.05 mm/rev for all the 5 mm diameter holes, performed with the help of a 5 mm diameter K20 tungsten carbide Brad point drill (see Figure 4) according to some of the papers referred above and recommendations of tool manufacturer. This drill geometry, due to its particular geometry, causes the tensioning of the fibres prior to the cut, enabling a clean surface. During the drilling operation, the thrust forces and torque were continuously monitored by using a KISTLER (Winterthur, Switzerland) 9171A rotating 4-component cutting force dynamometer associated with a computer for data collection. To avoid wear effects, the tool was replaced after every 4 plates were drilled.

The results considered as pertinent for this study were the thrust force during drilling and the flexural resistance of the drilled plates. The 4-point flexural test was performed at the Technological Testing Laboratory (LET) in ISEP, as described in the next section. For a complete and unbiased comparison of the results, a 3-point flexural test was performed on a similar set of non-drilled plates in UFJSR. The goal from the authors was to confirm equivalent results and conclusions from an experimental study carried out on carbon/epoxy plates [32]. After drilling, and before mechanical testing, the use of an NDT method such as enhanced radiography or another method was extensively referred to, in order to assess the delamination extension [33,34,35]. In the case of this work, NDT assessment was not performed due to the small thickness of the plates. So, it was considered that larger thrust forces would cause a larger delamination extension.

The final step of the experimental pipeline was the completion of a 4-point flexural test with the hole located at the centre of the coupons with a dimension of 100 × 30 × 1 mm^3^ and a test speed (*ν*) equal to 9.2 mm/min, according to the conditions set in the ISO 14125:1998 standard [36], see Equation (2). In the 4-point bending flexural test there are no shear forces in the area between the two loading pins, only a constant bending moment. These flexural tests were carried out using a Shimadzu (Kyoto, Japan) AG-X Plus 100 kN universal testing machine.

(2) $$v=\frac{\varepsilon^{\prime }\times L^{2}}{4.7\times h}$$

where *ε′* is a strain rate of 0.01 (or 1% per minute), *L* (distance between the supports) is a span equal to 66 mm and *h* the thickness of the coupon, equal to 1 mm.

The same standard was used for deriving the corresponding maximum flexural strength for each coupon, see Equation (3), considering a span of 66 mm (*L*) and a distance between the two arms of the anvil of 22 mm.

(3) $$\sigma_{f}=\frac{F\times L}{b\times h^{2}}$$

where *F* is the load, in N, *b* is the coupon width, in mm and *L* and *h* are as in Equation (2).

As referred to above, a 3-point test was also performed on a set of similar plates, without drilling, in the laboratories of UFSJR, Brazil. These tests were performed using a Shimadzu (Kyoto, Japan) 100 kN universal testing machine, following ASTM D790:2017 [37], with a crosshead speed of 1 mm/min.

## 3. Results and Discussion

Considering the large number of coupons and respective results to present, the experimental results are summarised in Table 3, both for drilling thrust forces and 4-point flexural strength of the drilled plates and for the 3-point flexural test of undrilled plates. Then, for further discussion and analysis, the results are grouped in 8 subsets according to the following criteria: matrix/reinforcement volume fraction; type of particle; stacking sequence, as previously defined.

### 3.1. Thrust Force during Drilling

The results of the thrust force monitoring were collected during the sequence of the six consecutive holes drilled in the central region of each plate (Figure 2). For comparison purposes, the maximum thrust force was the value considered, although the variation in this result was not significant along the different plates used in this experimental work. This outcome can be explained by the thickness of the plates, equal to 1 mm, and consequent low mechanical resistance to the drill cutting and breakthrough action as well as the option for conservative values of the drilling parameters.

**Figure 5:**
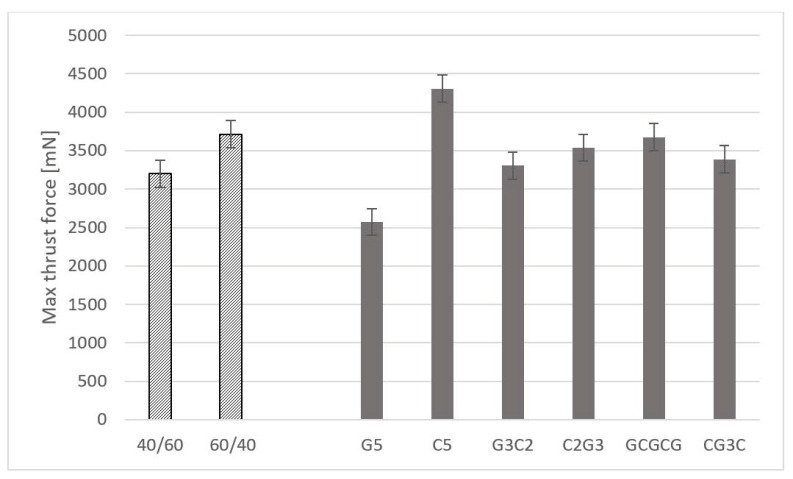
Maximum thrust force for different volume fractions and stacking sequences.

The values presented in Table 3 are the average of the maximum thrust force registered for each condition of the plates (see Table 2 for identification). Due to the range of values observed during drilling, results are presented in mN instead of N. These values are then aggregated considering the grouping criteria in the subsets referred to above. For the fibre/reinforcement volume fraction subsets, an increase of 16% on the average of the maximum thrust force was noted when decreasing the volume fraction from 60% to 40%, as in the two left bars in Figure 5. This outcome can be related to the enhanced bulk effect of the matrix by assuring the mechanical link of the fibres and particles against the piercing action of the drill bit. Regarding the stacking sequence effect, the higher results of the thrust force were obtained for the C5 plates (carbon reinforced), as expected, with an increase of around 70% when compared with G5 plates (see the right-side bars in Figure 5). Interesting to mention is the effect of hybrid composites, combining two layers reinforced with carbon and three layers reinforced with glass (G3C2 or C2G3). In this case, there is a positive effect on the thrust force and a value that may be seen as a balanced one, combining lower costs with a reasonable mechanical strength, here evidenced by the thrust force. Particularly, the GCGCG plates, alternating glass-reinforced layers with carbon-reinforced layers seem promising, showing a maximum thrust force decrease of only 11% when compared to the C5 plates. These features can be compared with the results of the apparent density presented in [25], as this outcome is mainly dependent on the fibre arrangement and the slight increase in the apparent density caused by the particle inclusion, ranging from 1.7 to 2.8 g/cm^3^, whereas the glass fibre or the carbon fibre densities are, respectively, 2.65 and 1.77 g/cm^3^.

**Figure 6:**
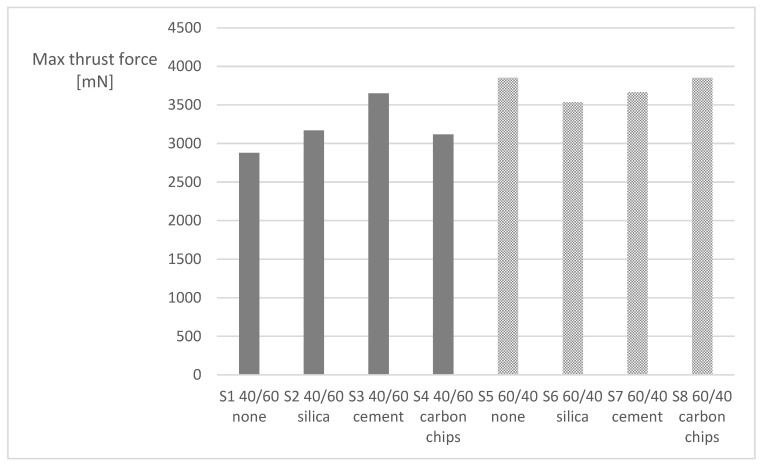
Maximum thrust force for sets S1 to S8 (as defined in Table 2).

Finally, when considering the subsets S1 to S8, according to the particle type as defined in Table 2 a similar outcome can be seen, as the decrease in volume fraction has the same effect of raising the maximum thrust force needed to drill the plates by 17% in a global average (see Figure 6). It has to be noted that, due to the small thickness of the plates, the rise and drop of the thrust forces during drilling occurs in a short time, approximately one second; therefore, the peak force is seen as a good result for comparison of the plate behaviour during the drilling operation. Adding particles heightened the thrust force, independently of the particle type, for the plates with 60% volume fraction. For the plates with 40% volume fraction, the effect of added particles was not noticeable. It is interesting to note that a higher thrust force value was obtained for the plates without particles. This outcome shows that there is, on average, a positive effect on the reduced use of reinforcement fibres and that the use of particles can compensate for, in a limited but positive way, this reduction. Thus, the use of waste particles can be helpful in the mechanical characteristics of the plate under controlled conditions. As can be observed in Table 3, there was a moderate increase in the thrust force, meaning more resistance of the drilled plates to the punching action of the drill, more pronounced on the 60/40 plates. This aspect deserves further analysis, which is beyond the scope of the present work.

### 3.2. Flexural Testing

**Figure 7:**
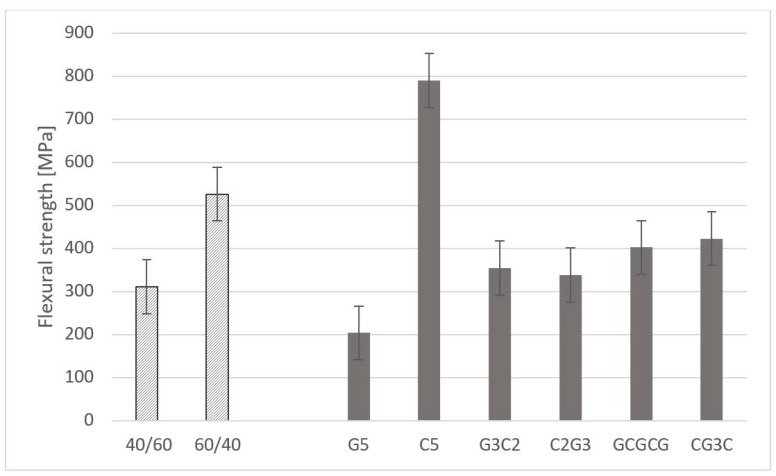
Flexural strength for different volume fractions and stacking sequences.

**Figure 8:**
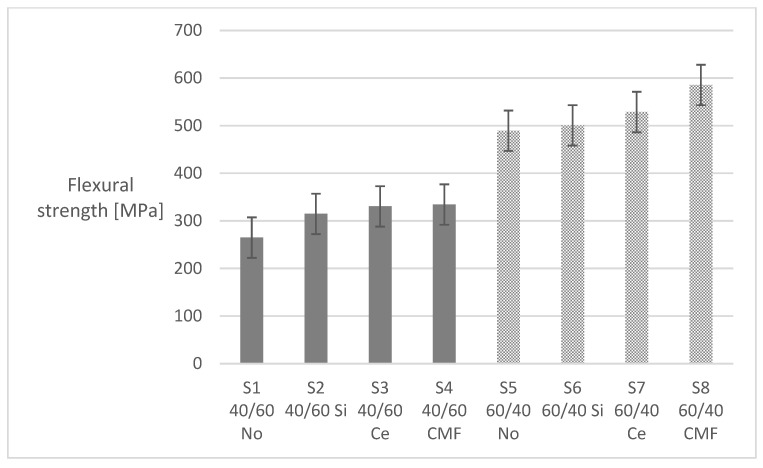
Flexural strength for sets S1 to S8 (as defined in Table 2).

The 4-point flexural tests were performed on the drilled plates, with a 5 mm hole at the geometric centre of the plate (see Figure 2) and the test coupons were numbered 1 and 2 for each of the 96 plates. According to the experimental conditions described in Table 2 and in Figure 2, four coupons of the same type were tested for each of the 48 formulations. The aggregated results considering the defined subsets are presented in Figure 7 and Figure 8. For these tests, the goal was to observe the outcome of the drilled plates when loaded in bending, as the existence of a drilled hole causes a reduction in the flexural strength of the plates, independently of the damage caused by the machining operation [32]. Even though the results of the 3-point flexural test performed in Brazil are presented in Table 3, they are considered mainly for comparison purposes, as the discussion on the outcomes is focused on the 4-point flexural test. Note that the major trends of the results, as discussed below, are essentially identical. As expected, due the major differences between the two test procedures, some significant dissimilarities were observed in the results presented in Table 3. For 3-point flexural tests, the plates were not previously drilled, and the development of both stresses is different due to the test setup. In 3-point flexural testing the peak stress is at the coupon mid-point and stress is reduced elsewhere. In 4-point flexural testing, the peak stress is exerted along an extended region of the coupon, hence exposing a larger length of the material, namely between the two inner points of the load application.

As expected, the higher results in both test procedures were mostly observed when testing C5 plates, followed by the plates with carbon outer plies (G3C2, C2G3, CG3C). Some discrepancies are noteworthy when comparing 3-point with 4-point flexural test results, most of them on the plates with a 60/40% volume fraction with particles—plates 31 to 48. This outcome may be caused by some interaction of the particles with the reinforcement fibres, especially in non-drilled plates. For the subsets of 40/60% plates, the only odd result was verified when silica particles were added. These results will deserve further study to understand the possible interaction effects and their causes.

For the matrix/reinforcement volume fraction, the same bulk effect on the matrix/fibre content was observed, with an increase of about 70% on the flexural strength (see the left side bars in Figure 7). The most noteworthy effect is on the stacking sequence, as carbon plies have a definitive impact on the flexural strength, returning higher values for the plates that had carbon plies as outer plies (CGGGC)—see the rightmost bar in Figure 7—and a noteworthy improvement of almost 90% in comparison with the average values of the 4-point flexural strength for the C5 plates (carbon-reinforced only). This result can be explained by the superior mechanical strength of carbon fibres when compared to glass fibres. Therefore, the use of hybrid composites should bear in mind the positive effect of having carbon-reinforced outer plies if parts are supposed to bear bending loads. The inner plies reinforced with glass fibre do not show a negative effect on the mechanical features and make the hybrid composite less expensive. As already referred to in Section 3.1, these results have to be related to the outcomes of the apparent density presented in [25], concerning the effect of the stacking sequence and particle type on the density of composites, and thus on their physical properties.

Looking at the results of the 4-point flexural test ordered by the subsets S1 to S8, Figure 8, the higher results in flexural testing were observed for the plates with the higher matrix content, with an overall increase of 70%. This outcome is in accordance with the previous results presented in this work.

There is also an enhancing impact on the flexural strength when comparing the flexural strength of the plates with no particle with the plates with some particle added, independently of the particle type. On average, this increase can reach 20% in plates with less fibre content (40%). This increase is more evident when carbon microfibres were added. Further studies are needed to more deeply understand this effect on hybrid plates. This result highlights that the reuse of waste particles is possible, not causing any prejudicial effect on the mechanical properties of the composite plates.

## 4. Conclusions

In this work, hybrid composites reinforced with glass and carbon fabrics and three different micro-inclusions: silica particles, recycled carbon fibre powder and cement were assessed regarding their machinability and flexural resistance. The stacking sequence of glass and carbon plies was also varied, enabling a total of 48 different formulations. The following results from the experimental work can be presented.

The different ratios of matrix/reinforcement have consequences on the thrust force needed for drilling but also on the flexural strength of the hybrid plates.

The plates reinforced with carbon in the outer plies had higher flexural resistance, the C5 plates (carbon only) being the ones with the highest values, more than 20% above the values observed with other stacking sequences, on average.

These results can be related to the changes in the apparent density of the composites, due to the diverse densities of the reinforcement fibres.

Adding particles (9% weight), regardless of the type, increased the mechanical strength of the plates, as observed by the values of the drilling thrust force and flexural strength. For the drilling thrust force, however, this effect was less noted when the amount of reinforcement fibres rose from 40% to 60%. For the flexural strength there was a positive effect on the inclusion of particles, with the most noticeable result being when carbon microfibres were added. Again, these results are related to the slight change in the apparent density of composites with these micro-inclusions.

Looking closely at the effect of the inclusion of the different particles, some increase in the flexural strength was noted in some plates, but the main point is that these inclusions do not cause a decrease in the flexural strength, and thus can contribute to the use of less fibre, successfully replacing them in some content.

These formulations of hybrid composite materials, including carbon microfibres or other particles like silica or cement, can contribute to the reduction of waste, to environmental objectives and to the saving of raw materials like reinforcement fibres, enabling a circular economy life cycle for these composites.

## Figures and Tables

**Table 1:** Matrix planning: factors and experimental levels.

Factors	Experimental Levels	Short Designation
Stacking sequence	Glass fibres only	G5
	Carbon fibres only	C5
	Glass/Glass/Glass/Carbon/Carbon	G3C2
	Carbon/Carbon/Glass/Glass/Glass	C2G3
	Carbon/Glass/Glass/Glass/Carbon	CG3C
	Glass/Carbon/Glass/Carbon/Glass	GCGCG
Volume fraction (wt%)	40/60	-
	60/40	
Particle type	No particle	No
	Silica, 9% wt, 2.7 g/cm^3^	Si
	Cement, 9% wt, 2.8 g/cm^3^	Ce
	Recycled carbon microfibres, 9% wt, 1.7 g/cm^3^	CMF

**Table 2:** Experimental conditions and subsets.

Subset	Plate #	%Matr/Reinf (vol)	Particle Type	Stacking Sequence
S1	1	40/60	No	G5
	2	40/60	No	C5
	3	40/60	No	G3C2
	4	40/60	No	C2G3
	5	40/60	No	GCGCG
	6	40/60	No	CG3C
S2	7	40/60	Si	G5
	8	40/60	Si	C5
	9	40/60	Si	G3C2
	10	40/60	Si	C2G3
	11	40/60	Si	GCGCG
	12	40/60	Si	CG3C
S3	13	40/60	Ce	G5
	14	40/60	Ce	C5
	15	40/60	Ce	G3C2
	16	40/60	Ce	C2G3
	17	40/60	Ce	GCGCG
	18	40/60	Ce	CG3C
S4	19	40/60	CMF	G5
	20	40/60	CMF	C5
	21	40/60	CMF	G3C2
	22	40/60	CMF	C2G3
	23	40/60	CMF	GCGCG
	24	40/60	CMF	CG3C
S5	25	60/40	No	G5
	26	60/40	No	C5
	27	60/40	No	G3C2
	28	60/40	No	C2G3
	29	60/40	No	GCGCG
	30	60/40	No	CG3C
S6	31	60/40	Si	G5
	32	60/40	Si	C5
	33	60/40	Si	G3C2
	34	60/40	Si	C2G3
	35	60/40	Si	GCGCG
	36	60/40	Si	CG3C
S7	37	60/40	Ce	G5
	38	60/40	Ce	C5
	39	60/40	Ce	G3C2
	40	60/40	Ce	C2G3
	41	60/40	Ce	GCGCG
	42	60/40	Ce	CG3C
S8	43	60/40	CMF	G5
	44	60/40	CMF	C5
	45	60/40	CMF	G3C2
	46	60/40	CMF	C2G3
	47	60/40	CMF	GCGCG
	48	60/40	CMF	CG3C

**Table 3:** Experimental results.

Subset	Plate #	Max Thrust (mN)	4P Flex Strgth ^1^ (MPa)	3P Flex Strgth ^1^ (MPa)
S1	1	2700	173	219
	2	3313	470	333
	3	2307	147	225
	4	2200	228	236
	5	3352	265	310
	6	3408	306	344
S2	7	1973	122	229
	8	3623	573	391
	9	2861	291	305
	10	3768	236	366
	11	3601	360	352
	12	3185	306	357
S3	13	2536	87	227
	14	4843	715	317
	15	3846	212	233
	16	3310	289	244
	17	3714	331	302
	18	3644	349	319
S4	19	2110	79	214
	20	3318	509	328
	21	3220	275	246
	22	3473	345	250
	23	3428	394	366
	24	3157	404	370
S5	25	2644	264	235
	26	4874	1031	480
	27	4303	466	478
	28	4021	426	402
	29	3553	396	396
	30	3722	353	235
S6	31	2989	241	295
	32	4973	1029	500
	33	2940	418	447
	34	3678	333	273
	35	3634	444	440
	36	3003	539	307
S7	37	2742	331	255
	38	4752	957	496
	39	3404	491	466
	40	3708	385	395
	41	3819	478	408
	42	3559	530	347
S8	43	2877	332	250
	44	4756	1036	504
	45	3563	534	406
	46	4150	467	381
	47	4318	548	396
	48	3440	596	336

^1^ flex strgth = flexural strength.

## Data Availability

Derived data supporting the findings of this study are available from the corresponding author upon request.
